# Selenium Discrepancies in Fetal Bovine Serum: Impact on Cellular Selenoprotein Expression

**DOI:** 10.3390/ijms25137261

**Published:** 2024-07-01

**Authors:** François Parant, Fabrice Mure, Julien Maurin, Léana Beauvilliers, Chaïma Chorfa, Chaymae El Jamali, Théophile Ohlmann, Laurent Chavatte

**Affiliations:** 1 Service de Biochimie et Biologie Moléculaire, Laboratoire de Biologie Médicale Multi-Sites (LBMMS), Hôpital Lyon-Sud-Hospices Civils de Lyon, 69495 Pierre-Bénite, France; francois.parant@chu-lyon.fr (F.P.); julien.maurin@chu-lyon.fr (J.M.); leana.beauvilliers@orange.fr (L.B.); 2 Centre International de Recherche en Infectiologie (CIRI), 69007 Lyon, France; fabrice.mure@ens-lyon.fr (F.M.); chaima.chorfa@outlook.fr (C.C.); chaymae.el-jamali@etu.univ-lyon1.fr (C.E.J.); 3 Institut National de la Santé et de la Recherche Médicale (INSERM), Unité U1111, 69007 Lyon, France; 4 Ecole Normale Supérieure de Lyon, 69007 Lyon, France; 5 Division Recherche, Université Claude Bernard Lyon 1 (UCBL1), 69008 Lyon, France; 6 Centre National de la Recherche Scientifique (CNRS), Unité Mixte de Recherche 5308 (UMR5308), 69007 Lyon, France

**Keywords:** triple quadrupole ICP-MS, selenium concentration, selenoprotein, GPX1, GPX4, TXNRD1, selenocysteine

## Abstract

Selenium is an essential trace element in our diet, crucial for the composition of human selenoproteins, which include 25 genes such as glutathione peroxidases and thioredoxin reductases. The regulation of the selenoproteome primarily hinges on the bioavailability of selenium, either from dietary sources or cell culture media. This selenium-dependent control follows a specific hierarchy, with “housekeeping” selenoproteins maintaining constant expression while “stress-regulated” counterparts respond to selenium level fluctuations. This study investigates the variability in fetal bovine serum (FBS) selenium concentrations among commercial batches and its effects on the expression of specific stress-related cellular selenoproteins. Despite the limitations of our study, which exclusively used HEK293 cells and focused on a subset of selenoproteins, our findings highlight the substantial impact of serum selenium levels on selenoprotein expression, particularly for GPX1 and GPX4. The luciferase reporter assay emerged as a sensitive and precise method for evaluating selenium levels in cell culture environments. While not exhaustive, this analysis provides valuable insights into selenium-mediated selenoprotein regulation, emphasizing the importance of serum composition in cellular responses and offering guidance for researchers in the selenoprotein field.

## 1. Introduction

Selenium is an essential trace element in mammals which plays a pivotal role in redox biology. Its deficiency has been extensively associated with an elevated risk of various pathologies, including cancers, neurological diseases, cardiovascular and endocrine disorders, and infectious ailments [1,2,3,4,5,6]. This essential role primarily stems from its presence as selenocysteine (Sec) within selenoproteins, which is unique as it is encoded by a specific UGA codon, making it the 21st amino acid [2,7,8,9]. Sec stands apart from other proteinogenic amino acids due to its unique synthesis pathway involving a specialized tRNA, dedicated recoding factors, and its encoding by a UGA codon, normally used as a stop signal [7,10,11,12,13,14,15,16]. Most selenoproteins function as oxidoreductases and hold significant importance for human health. The human genome contains twenty-five selenoprotein genes, the regulation of which primarily depends on the bioavailability of selenium, whether derived from dietary sources or cell culture media [17,18,19,20,21,22,23,24,25,26,27]. This selenium-dependent control adheres to a specific hierarchy, whereby “housekeeping” selenoproteins maintain constant expression at the expense of “stress-regulated” counterparts responsive to selenium level fluctuations [11,28,29]. Among these selenoproteins, glutathione peroxidases (GPXs) and thioredoxin reductases (TXNRDs) stand as well-characterized representatives [30]. GPXs participate in antioxidant defenses by reducing a wide array of peroxides, employing glutathione (GSH) as a cofactor. The expression of most GPXs is highly sensitive to selenium level variations [25,31,32]. Among the GPXs, GPX1 and GPX4 have been extensively characterized due to their ubiquitous expression. In most cells, GPX1 exhibits much greater reactivity than GPX4 [31,32]. Thus, in HEK293 cells, GPX1 is ten times more sensitive to selenium variations in the culture medium than the protein GPX4 [25]. In primary CD4 T cells purified from four different donors, as well as in the Jurkat and SupT1 lymphocyte cell lines, GPX1 expression is two to three times more selenium-sensitive than GPX4 [24]. On the other hand, TXNRDs are NADPH-dependent reductases that govern the redox balance of various substrates, encompassing proteins and small selenium- and sulfur-containing molecules. TXNRDs are considered housekeeping members since they are maintained even in selenium deficiency at the expense of others [25,32,33,34]. This distinctive regulation of GPXs in comparison to TXNRDs has been observed across various cellular models, including HEK293 [25], and extended to other selenoproteins, although the mechanisms remain enigmatic [2,7,8,11].

**Figure 1:**
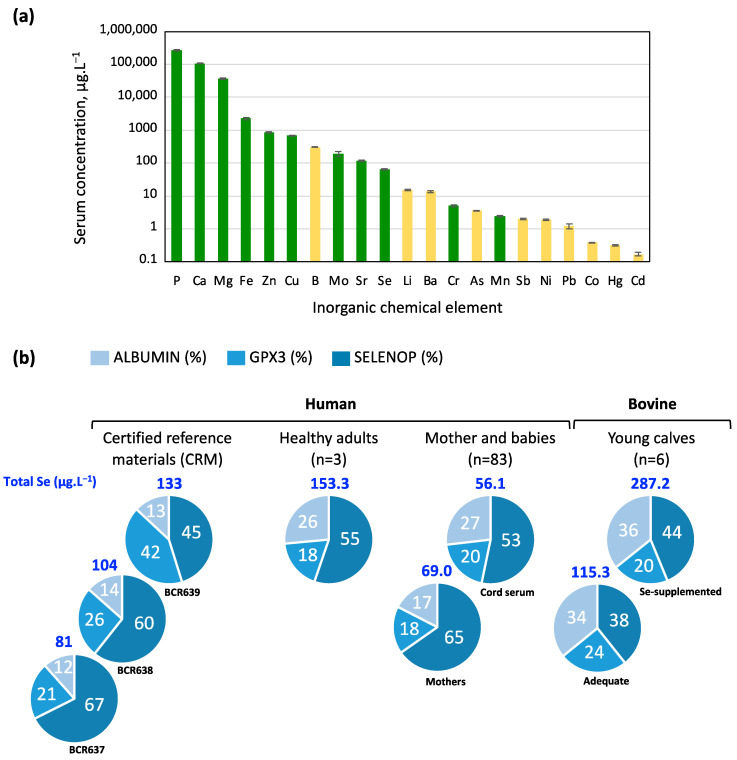
Elemental- and selenium-related composition of the serum as documented in the literature. (**a**) Concentration levels of toxic, neutral, and essential elements measured in the serum of lactating cows, detectable by multielement ICP-MS, adapted from [36]. The data are depicted as mean values ± standard error, utilizing a logarithmic scale, obtained from a random selection of 20 cows out of the 188 present on the farm situated in Northern Spain [36]. Essential elements are depicted in green bars, while the remaining elements are shown in yellow. Phosphorus (P), calcium (Ca), magnesium (Mg), iron (Fe), zinc (Zn), copper (Cu), boron (B), molybdenum (Mo), strontium (Sr), selenium (Se), lithium (Li), barium (Ba), chromium (Cr), arsenic (As), manganese (Mn), antimony (Sb), nickel (Ni), lead (Pb), cobalt (Co), mercury (Hg), and cadmium (Cd). (**b**) Pie charts depict selenium distribution among serum proteins in various contexts, with total selenium concentration indicated above each chart in blue. The various studies concerned human-certified reference materials [43], healthy human adults [41], human serum at birth [44], and young calves under different selenium diets [42]. Selenium distribution was assessed using ICP-MS coupled with various separation methods. Total selenium concentration is denoted above each chart in blue, and the percentage of each part is indicated in white on the pie chart.

This selenium-dependent regulation of the selenoproteome can be replicated in cultured cells where the growth medium is supplemented with selenium of either organic or inorganic form. Supplementation with radioactive or stable isotopes of selenium can also be employed to trace its incorporation into selenoproteins [25,35]. However, this can be achieved using fetal bovine serum (FBS) that is inherently deficient in selenium as FBS serves as a source of necessary nutrients, growth factors, and hormones for cell growth. In FBS, selenium is one of the essential trace elements detected with the lowest concentration, as illustrated in Figure 1a [36]. The selenium concentration in FBS has long been overlooked, as it was believed to be relatively consistent across various sources and suppliers. However, this is far from the truth, and there are significant variabilities that can impact the physiological processes of cultured cells. Karnelius et al. cautioned that this variability may influence redox-regulated gene expression [37]. In some laboratories, researchers conduct thorough evaluations of various FBS sources to find a serum that consistently fulfills their specific cell culture requirements, ensuring reproducibility and reliability in experimental outcomes prior to making large-scale purchases. Therefore, one of the analyses currently undertaken by our team involves measuring the total selenium concentration present in the FBS. Several methods are available, but the most accurate currently relies on elemental measurement using triple quadrupole inductively coupled plasma mass spectrometry (TQ ICP-MS) [38].

In human serum, as in all mammals, selenium exists in various forms. It is present in relatively low amounts in its free form. Mostly, it is either incorporated into the selenoproteins GPX3 and SELENOP or bound to albumin. Within selenoproteins, it exists in the form of the amino acid selenocysteine, whereas it occurs in an inorganic form when bound to albumin [39,40]. These three proteins constitute the majority of selenium present in serum. There is a significant technological challenge in assessing the ratio between these very different forms [41,42,43,44,45,46]. As illustrated in Figure 1a, even when utilizing cutting-edge analytical strategies combined with ICP-MS, there are discrepancies among studies [41,42,43,44]. Nonetheless, it appears that, for a given method, the relative distribution between albumin, SELENOP, and GPX3 remains relatively stable, regardless of whether the human serum contains high or low selenium levels [41,42,43,44]. In other words, these three proteins appear to be reliable markers of selenium status, as they consistently respond to variations in serum levels.

In cellular biology, there are several methods for measuring the incorporation of selenium into intracellular selenoproteins [47,48]. The first involves detecting its presence in Western blot using a specific antibody, yet we are dependent on the availability and quality of the antibodies. Additionally, there are assays for specific enzymatic activities of GPXs and TXNRDs. Among these methods, for GPX1, the immunological approach proves to be the most sensitive compared to ICP-MS or enzymatic activity measurements [47]. Another strategy involves measuring the recoding activity of the UGA codon to selenocysteine using a luciferase reporter. In this case, luciferase activity is only detected upon the insertion of a selenocysteine residue. This system is extremely sensitive, and its measurement can span several logarithmic scales using luminescent reagents [49].

Selenium represents one of the essential trace elements found at the lowest concentrations in the blood (Figure 1a). In humans, it is typically present at average values close to 100 µg.L^−1^, although significant disparities exist worldwide [50,51,52,53]. Indeed, serum selenium concentrations range from 20 µg.L^−1^ in selenium-deficient regions of China to an average of 122 µg.L^−1^ in the USA, with values exceeding 1000 µg.L^−1^ observed in the Inuit population of Greenland. In bovine serum, selenium appears even lower, with an average of 62.2 ± 2.6 µg.L^−1^ (mean ± standard error) and values ranging from 39.3 to 93.6 µg.L^−1^ [36].

In the current study, we concurrently evaluated the influence of 12 different FBS from various batches and suppliers on the capacity of HEK293 cells to express specific selenoproteins or to incorporate selenocysteine by UGA recoding. First, we observed a significant disparity in selenium concentration among these different FBS samples. Subsequently, as previously described, GPX1 and GPX4 appeared more sensitive to selenium variations than TXNRD1. A highly significant correlation was evident between the selenium level in the medium and the expression levels of GPX1 and GPX4. Enzymatic activity measurements of GPX and TXNRD were consistent with the data obtained from Western blot analyses. Ultimately, the luciferase activity obtained from cells stably expressing this reporter showed a clear correlation with selenium levels, providing a rapid, reliable, and precise tool for determining selenium levels and bioavailability in the culture medium.

## 2. Results

### 2.1. Selenium Levels in FBS Greatly Vary from Suppliers and Lot Number

**Figure 2:**
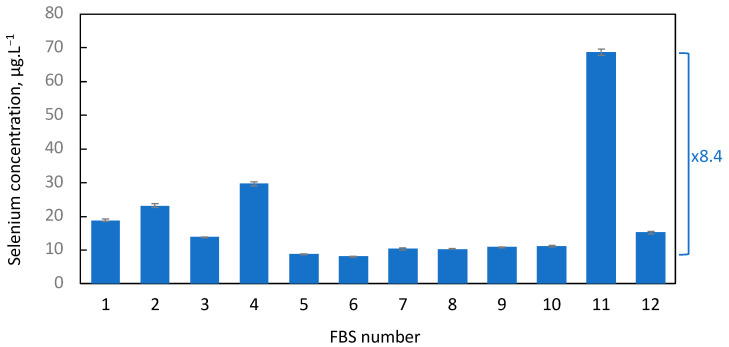
Variation in selenium concentration across the 12 different FBS samples analyzed in this study and listed in Table 1. TQ ICP MS was used in mass shift mode using O_2_ as a reaction gas to determine the levels of selenium. Each sample was analyzed in triplicate and represented as means ± S.D. The difference between the lowest and highest values is indicated by a blue bracket, with the corresponding fold-change factor beside it.

We analyzed selenium concentration in 12 commercial FBS samples from different companies and batches, see Table 1. The method employed here is dedicated to measuring the most abundant selenium isotope (^80^Se) using triple quadrupole ICP-MS after reaction with oxygen gas. By utilizing this method, we overcome interferences from argon dimers originating from the plasma gas while measuring the most abundant isotope present at 49.61%. Under these conditions, the limits of detection (LOD) and quantification (LOQ) are 0.18 and 0.40 µg.L^−1^, respectively, for serum samples. With this technology, we achieve relatively low serum concentrations in our samples. As depicted in Figure 2, selenium concentrations in FBS were usually low (average 19.1 ± 16.9 µg.L^−1^) and characterized by a large inter-batch and inter-supplier variability, with extreme values of 8.14 ± 0.06 µg.L^−1^ and 68.72 ± 1.01 µg.L^−1^ (mean ± S.D.) for FBS #6 and FBS #11, respectively. The ratio between these two FBS corresponds to an 8.4-fold change.

### 2.2. Impact of FBS Selenium Levels on the Expression of GPX1, GPX4, and TXNRD1 in HEK293 Cells

**Figure 3:**
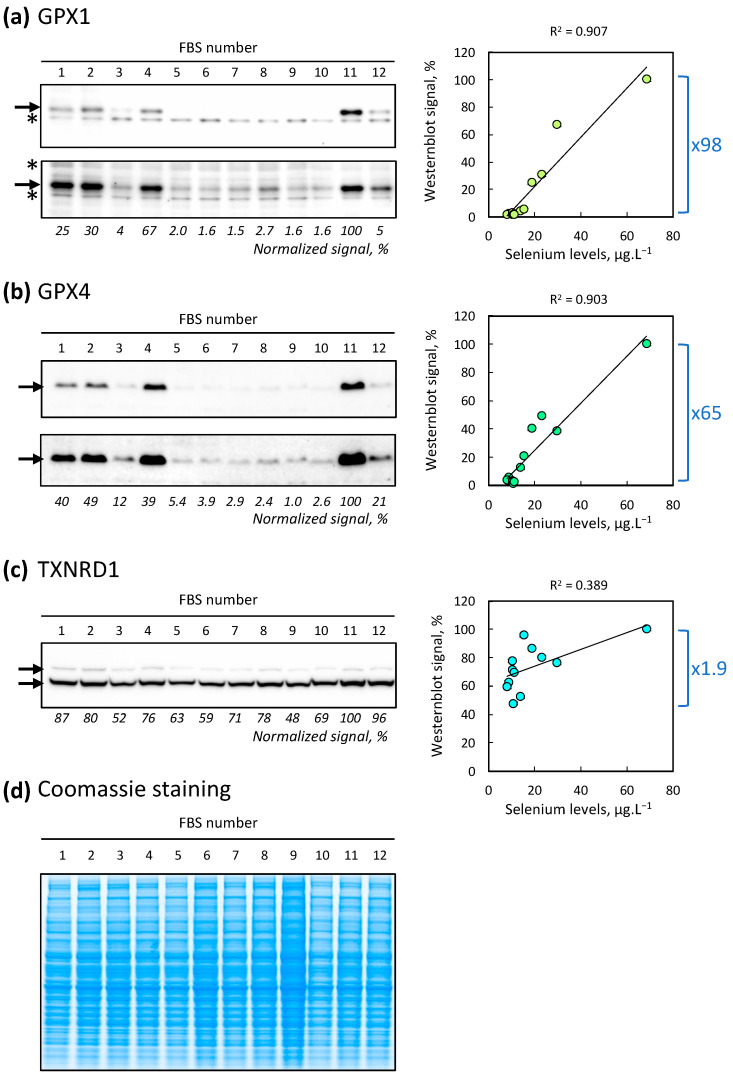
Variation of intracellular selenoprotein expression levels depending on the serum used, visualized via Western blot analysis. HEK293 cells were cultured in the presence of each FBS variant listed in Table 1 and harvested after three days of growth. Cellular extracts were subsequently separated on protein gels and subjected to Western blot analysis to determine the levels of GPX1 (**a**), GPX4 (**b**), and TXNRD1 (**c**). (**d**) One gel was stained with InstantBlue Coomassie staining solution and used for signal intensity normalization. For GPX1 and GPX4, an additional, more exposed gel is also presented to confirm the presence of signal at a lower level. The arrows indicate the migration of the selenoproteins. Non-specific bands are indicated by stars. The relative protein level intensity is indicated as a percentage of the strongest signal across the entire blot at the bottom of each image. On the right side of each blot, a graph depicting the Western blot signal intensity as a function of serum selenium concentration is displayed. A linear regression line is shown along with its Pearson linear determination coefficient (R^2^). For each graph, the differences between the lowest and highest values in the Western blot signal are indicated by a blue bracket, with the corresponding fold-change factor beside it.

The GPX family is well known to be highly responsive to variations in selenium levels in the culture medium or its availability in the body [17,18,19,20,21,22,23,24,25]. In our present study, where we cultured HEK293 cells with culture media containing 12 different FBS samples for 72 h, we observed a variation in the expression levels of GPX1 and GPX4 in cellular extracts, while that of TXNRD1 seemed relatively unaffected (Figure 3). The differences are such that a slightly longer exposure is needed to confirm the presence of GPX1 and GPX4 proteins under conditions where they are expressed to a lesser extent. For all three selenoproteins studied, the maximum expression level is achieved with FBS #11, which has the highest selenium concentration. It is also noted that several FBS conditions result in GPX1 and GPX4 levels at least 25 times lower.

Although Western blots remain semi-quantitative methods, we attempted to ascertain if there is a correlation between selenium concentrations in the culture medium and selenoprotein expression levels (right panels in Figure 3). In all three cases, we observed a positive correlation. However, only for GPX1 and GPX4, the Pearson linear determination coefficient (R^2^) exceeds 0.9, indicating a relatively strong correlation between selenium levels and selenoprotein expression.

In conclusion, the selenium level in the culture medium, even if its concentration varies only by a factor of about 8, influences the expression of the studied GPXs nearly a hundredfold. Our results also confirm that TXNRD1 is often maintained at the expense of GPX1 and GPX4.

### 2.3. Impact of FBS Selenium Levels on the GPX and TXNRD Enzymatic Activities in HEK293 Cells

The levels of certain selenoproteins, such as GPXs and TXNRDs, can also be assessed by measuring their enzymatic activities. For these proteins, catalytic activity is exclusively dependent on the presence of selenocysteine in the catalytic site. Similar to the protein levels observed by Western blot, the enzymatic activity of GPXs is much more sensitive to selenium variations than that of TXNRDs in most cell lines [25]. Regarding GPX activity, it is noteworthy that GPX1 is ten times more efficient than GPX4 in reducing tert Butyl hydroperoxide (t-BHP) substrate [54]. Thus, in our cellular extracts, we primarily measure the variation related to GPX1, which is the most abundant in HEK293 cells [25]. 

**Figure 4:**
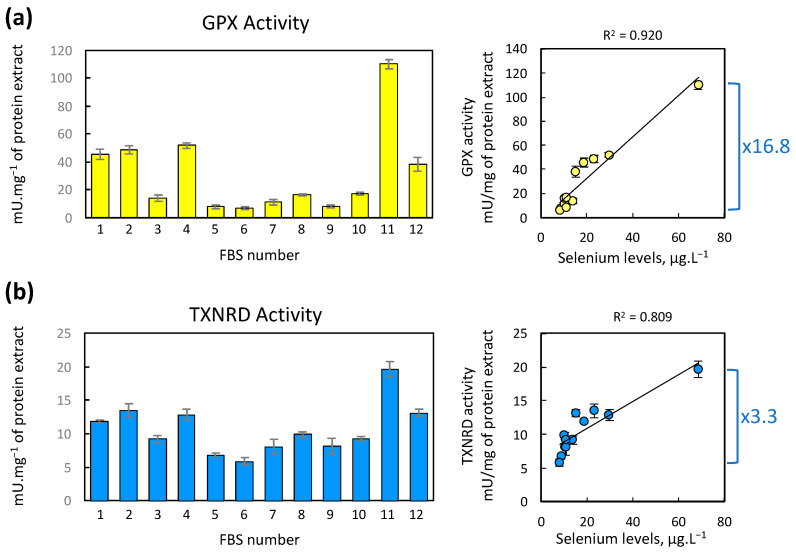
Evolution of intracellular GPX and TXNRD activity as a function of the FBS used. Cellular extracts from HEK293 cells cultured in the presence of each FBS variant were assessed for their GPX (**a**) and TXNRD (**b**) activities. The enzymatic activities are presented in histograms as the means of triplicates ± standard deviation. On the right side of each histogram, a graph illustrating the enzymatic activities in relation to serum selenium concentration is provided. The differences between the lowest and highest values of enzymatic activities are delineated by a blue bracket, accompanied by the corresponding fold-change factor.

In the present study, the same cellular extracts obtained above were also evaluated for their GPX and TXNRD activities, as shown in Figure 4, panels a and b, respectively. We found that the nature of the FBS stimulates a 16.8-fold change for GPX activity and a 3.3-fold change for TXNRD activity. Furthermore, in both sets of measurements, the ranking of enzymatic activities obtained with the different FBS is nearly identical and closely corresponds to the ranking of selenium concentrations detected by triple quadrupole ICP-MS. In both cases, it is FBS #6 and #11 that yield the lowest and highest values, respectively. To verify the quality of the correlation, we plotted the enzymatic activities as a function of selenium levels in FBS (panels to the right, Figure 4). In both cases, we observed a positive correlation that appears much more apparent with GPX activity than with TXNRD activity, as indicated by the Pearson linear determination coefficient (R^2^). In other words, GPX activity is highly sensitive to variations in selenium levels in the culture medium and can serve as a precise marker of this variation (R^2^ = 0.920).

### 2.4. Impact of FBS Selenium Levels on the UGA Recoding Activities as Measured by Luciferase Reporter Assay

**Figure 5:**
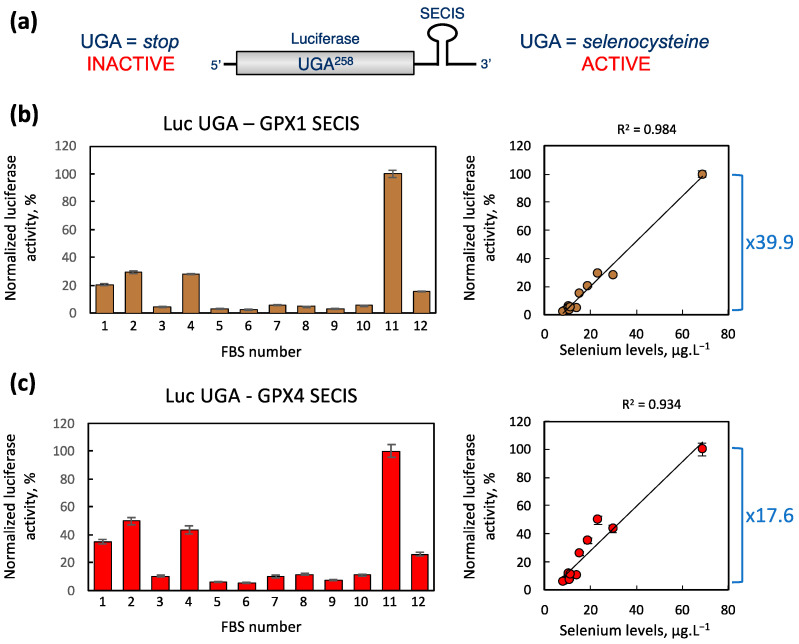
Use of stable HEK cell lines expressing luciferase reporters to assess the selenium content of FBS. (**a**) Schematic representation of the UGA-SECIS luciferase reporters. This system relies on the expression of a luciferase gene containing an in-frame UGA codon at position 258, downstream of which a minimal SECIS element is inserted. Two variants of this construct, featuring either GPX1 or GPX4 SECIS, were employed to establish stable cell lines [25,49]. Active luciferase enzyme is produced only when the UGA codon is recoded as selenocysteine and not when it is read as a stop codon. Cellular extracts from HEK293 cell lines expressing either Luc UGA—GPX1 SECIS (**b**) or Luc UGA—GPX4 SECIS (**c**) were cultured in the presence of each FBS variant and evaluated for luciferase activity. The luciferase enzymatic activities were normalized to protein concentration and then expressed relative to the strongest signal obtained with FBS#11, set as 100%. Data are presented in histograms as means of triplicates ± standard deviation. On the right side of each histogram, a graph illustrating the enzymatic activities in relation to serum selenium concentration is provided. The differences between the lowest and highest values of luciferase activities are depicted by a blue bracket, accompanied by the corresponding fold-change factor.

To confirm the correlation between selenoprotein expression and the selenium level in the culture medium, we used a reporter gene system adapted to measure the efficiency of selenocysteine insertion [25,49]. This system was developed and validated to study the translational regulation of selenoproteins in vivo (Figure 5a). The luciferase activity corresponds to the efficiency of UGA recoding into selenocysteine. HEK293 cells stably expressing Luc UGA-minimal selenocysteine insertion sequence (SECIS) GPX1 or Luc UGA-SECIS GPX4 facilitated the direct and rapid evaluation of selenocysteine insertion efficiencies in response to various stimuli, including selenium level variations. Cells were cultured with growth media containing the different FBS, and subsequently, cellular extracts were assayed for luciferase activities, normalized to protein concentrations, and expressed relative to the maximum activity measured, set as 100% (Figure 5b,c).

Consistent with our previous findings, the Luc UGA-GPX1 SECIS construct is slightly more sensitive to selenium variations than the Luc UGA-GPX4 SECIS construct. Similar to the earlier analyses in this study, FBS samples yielding minimum and maximum activities are #6 and #11, respectively. Stimulation factors of 39.9 and 17.6 are observed between these two FBS for these two cell lines, respectively. Interestingly, when plotting normalized luciferase values against the selenium concentrations of the FBS, we obtain very good correlations with Pearson linear determination coefficients (R^2^) of 0.93 and 0.98. These latest data confirm that these cell lines are excellent tools for rapidly screening and comparing selenium concentrations of different FBS batches.

## 3. Discussion

### 3.1. Selenium Levels in FBS Greatly Vary from Suppliers and Lot Numbers

The findings of our study confirm the hypothesis that commercially available FBS exhibits obvious variation in selenium concentration among different batches, markedly impacting the human selenoproteome profile in cell culture models. This protein family comprises members with diverse selenium regulation dynamics. Notably, “stress-regulated” selenoproteins, such as GPXs, are highly dependent on selenium status for expression, while “housekeeping” selenoproteins, exemplified by TXNRDs, show less regulation by selenium levels. This hierarchy of selenium utilization, incompletely understood in humans due to challenges in comprehensive selenoproteome detection, appears to be tissue- or cell-model-dependent. The speciation of selenium in serum, a considerable challenge, has been explored by various laboratories [41,42,43,44,45,46]. Separation strategies coupled with ICP-MS detection have aided in estimating selenium proportions in GPX3, SELENOP, and albumin (Figure 1). However, characterizing selenium metabolite proportions remains challenging. Our study utilized a highly precise ICP-MS triple quadrupole with oxygen reaction gas, revealing commercial FBS samples to be relatively selenium-deficient, with a mean concentration of 19.1 ± 16.9 µg.L^−1^ (mean ± S.D.), exhibiting an 8.4-fold difference between lowest and highest concentrations.

### 3.2. Selenium Levels in FBS Correlates with GPX1 and GPX4 Expression in Cells

In our investigation, we demonstrated that selenium concentration variation in the culture medium differentially influences selenoprotein expression in commonly used HEK293 cells. Notably, for most FBSs utilized, selenium content did not support optimal GPX1 and GPX4 expression. Almost half of the FBS samples yielded nearly undetectable levels of these selenoproteins by Western blot and enzymatic activity assays. However, overall analysis revealed a positive correlation between FBS selenium concentration and GPX1/GPX4 expression, both by Western blot and enzymatic activity assays. Conversely, TXNRD1 expression showed weak selenium dependence, affirming its “housekeeping” role.

FBS with low selenium levels can mimic selenium deficiency conditions, facilitating hierarchical selenoprotein regulation studies and exploring the bioavailability of selenium forms. Our work stresses the necessity of systematically monitoring FBS selenium concentration before conducting experiments on the role and regulation of selenoproteins. The method used for measuring total selenium in our study is highly precise and sensitive. By employing TQ ICP-MS with oxygen reaction gas, we ensured the accurate and reliable quantification of selenium levels, which is crucial for studying its effects on selenoprotein expression. This methodological strength adds robustness to our findings and underscores the reliability of our results.

Furthermore, we established that HEK293 cells stably expressing the luciferase UGA258 reporter coupled with a SECIS element provide a precise and rapid method for estimating FBS selenium concentration. The strong correlation between luciferase activity and FBS selenium concentration (Figure 5), attributable in part to luciferase’s broader dynamic range compared to Western blotting, supports this approach.

This extensive analysis offers significant insights into the regulation of selenoproteins mediated by selenium, underscored by the critical role of serum composition in cellular responses. Moreover, it provides essential guidance for researchers in the selenoprotein domain. Future work will include extending our analysis to other cell lines to validate and generalize our findings, addressing the limitation of using a single cell line in the current study. This approach will help to establish the broader applicability and significance of our results across different cellular contexts.

### 3.3. Selenium Bioavailability from FBS Is Mostly Supported by SELENOP

Regarding selenium forms’ biodisponibility in FBS for cells, SELENOP predominates as a major selenium transporter, particularly in blood plasma. While SELENOP is mainly hepatically produced, GPX3, also known as extracellular GPX, contributes to selenium delivery. SELENOP knockout mice exhibit reduced selenium levels in organs like the brain, kidneys, testes, and bones. The immunodepletion of SELENOP from FBS notably reduces GPX1, GPX4, and TXNRD1 expression in cell culture models, affirming SELENOP’s pivotal role in cellular selenium assimilation [55]. SELENOP uptake mechanisms involve three identified receptors: ApoER2 (LRP8), megalin (LRP2), and LRP1 [56]. Notably, LRP8 inactivation leads to a distinct lipid peroxidation-dependent cell death, ferroptosis, highlighting SELENOP’s essential role in cellular homeostasis [56].

## 4. Materials and Methods

### 4.1. Materials

HEK293T cell lines used in this study were obtained from American Type Culture Collection (ATCC, Manassas, VI, USA). Cell culture media, cell culture supplements, NuPAGE 4–12% bis–Tris polyacrylamide gels, 3-morpholinopropane-1-sulfonic acid (MOPS) sodium dodecyl sulfate (SDS) running buffer, were purchased from Life Technologies (ThermoFisher Scientific, Waltham, MA, USA). β-Nicotinamide adenine dinucleotide 2′-phosphate (NADPH), t-BHP, thioredoxin, L-GSH, glutathione reductase, 5,5′-Dithiobis 2-nitrobenzoic (DTNB), sucrose, dimethyl sulfoxyde (DMSO), 2,2′,2″,2′′′-(Ethane-1,2-diyldinitrilo)tetraacetic acid (EDTA), Triton X100, glycerol, and DL-Dithiothreitol (DTT) were purchased from Merck (Darmstadt, Germany). The luciferase assay reagent was purchased from Promega (Charbonnières, France). The microplate readers FLUOSTAR OPTIMA and LUMISTAR OPTIMA were from BMG Labtech (Champigny-sur-Marne, France). Antibodies were purchased from Abcam (Cambridge, UK) (GPX1, #ab108427, 1/1000 dilution; GPX4, #ab125066, 1/1000 dilution; TXNRD1, #ab124954, 1/10,000 dilution) and Merck (HRP-conjugated goat anti-rabbit IgG, #A6154, 1/40,000 dilution). A description of the different FBSs used in this study is shown in Table 1. The FBSs were either purchased or were trial samples offered by the manufacturer.

### 4.2. Triple Quadrupole ICP-MS Analysis

Levels of Se in FBS were determined by a triple quadrupole ICP-MS instrument (NexION 5000 from PerkinElmer (Waltham, MA, USA) with an ESI autosampler 2DXCi) in mass shift mode using O_2_ as reaction gas. Briefly, the first quadrupole (Q1) was set to transmit ^80^Se^+^. Potential interferents on the product ion (e.g., ^96^Zr^+^) were rejected. In the reaction cell (Q2), the reaction gas O_2_ was used to convert ^80^Se^+^ into ^80^Se^16^O^+^. The third quadrupole (Q3) was set to transmit *m/z* 96; other *m/z* were rejected (e.g., ^40^Ar^40^Ar^+^, ^160^Gd^++^, ^79^Br1H^+^). The standard and sample solutions were diluted to 1:50 with an aqueous mixture containing 0.5% *v/v* HNO_3_ and 1% *v*/*v*. butanol. Rhodium (^103^Rh) added on-line was used as internal standard. The method had an analytical measurement range of 0.5–200 µg.L^−1^. The intermediate imprecision ranged between 4.5% (mean Se concentration 39 μg.L^−1^) and 4.0% (178 μg.L^−1^). External quality assessment was provided through participation in a European “trace elements” quality control OELM (occupational and environmental laboratory medicine).

### 4.3. Cell Culture

Adherent cells (HEK293T) were grown and maintained in 75 cm^2^ plates in Dulbecco’s Modified Eagle Medium (D-MEM). Media were supplemented with 10% fetal calf serum, 100 μg.mL^−1^ streptomycin, 100 UI.mL^−1^ penicillin, and 2 mM L-glutamine. Cells were cultivated at 37 °C in humidified atmosphere containing 5% of CO_2_. Twelve culture media were prepared, each containing one of the 12 FBS samples at a concentration of 10%. A total of 1.5 × 10^6^ cells were seeded into 10 cm diameter dishes with 15 mL of medium. The cells were cultured for three days before being harvested and analyzed.

### 4.4. Protein Extraction and Analysis by Western Blot

After a wash with 1X PBS, cellular protein extracts were harvested with lysis buffer (25 mM Tris-HCl, pH 7.8, 2 mM DTT, 2 mM EDTA, 1% Triton X-100, and 10% glycerol). Then, protein concentrations were measured using the DC protein assay kit (Biorad) in microplate assays. Equal protein amounts (30 μg) were separated in 4–12% Bis-Tris NuPAGE Novex Midi Gels and transferred onto nitrocellulose membranes using the iBlot^®^ DRy blotting System. Membranes were probed with indicated primary antibodies and HRP-conjugated anti-rabbit antibodies. The chemiluminescence signal was detected using an ECL Select detection kit (GE Healthcare, Chicago, IL, USA) in the Chemidoc Imager (Biorad, Hercules, CA, USA). Data quantifications from chemilumesennce (Western blot) and colorimetric (Coomassie blue staining) images were performed with ImageLab Software (Biorad, Version 6.0.1).

### 4.5. GPX and TXNRD Enzymatic Assays

GPX and TXNRD activities were measured in enzymatic coupled assay as described in [32] with 30 μg of protein extracts. GPX enzymatic activities (U.mg^−1^) were measured with t-BHP substrate and expressed as nmol of glutathione/min·mg. TXNRD enzymatic activities (U.mg^−1^) were expressed as nmol of NADPH.min^−1^.mg^−1^.

### 4.6. Luciferase Assays

To analyze selenocysteine insertion efficiency in HEK293, we used luciferase-based reporter constructs [25,49]. Briefly, the SECIS elements from GPX1 and GPX4 were cloned downstream of a luciferase coding sequence, which was modified to contain an in-frame UGA codon at position 258 (Luc UGA/SECIS), as shown in Figure 5a. HEK293 cells stably expressing Luc UGA/GPX1 and Luc UGA/GPX1 SECIS were previously generated and validated [25,49]. After being grown in various FBS-containing media, cells were harvested, and the cellular extracts were assayed for luciferase activities by chemiluminescence (Luciferase assay systems, Promega, Charbonnières, France) in triplicate using a microplate reader FLUOstar OPTIMA (BMG Labtech). Luciferase activities were normalized over the protein concentrations of each extract and expressed arbitrarily relative to the maximum activity detected in experiment with FBS#11.

### 4.7. Graphical Illustrations and Data Analysis

Graphical illustrations (histograms and scatter plots) and linear regressions were developed and performed with Microsoft Excel for Mac 2019, version 16.78.3. Mean values were represented with error bars indicating the standard deviation (S.D.).

## Figures and Tables

**Table 1:** Description of the FBS used in this study.

FBS	Supplier (Distributor)	Supplier Location (City, State, Country)	Reference	Batch	Origin
#1	Gibco (Thermo Fisher Scientific)	Waltham, MA, USA	10-437-028	2230743RP	Mexico
#2	Gibco (Thermo Fisher Scientific)	Waltham, MA, USA	10-437-028	2286113RP	Mexico
#3	Eurobio Scientific	Les Ulis, France	CVFSVF00-01	S73136	South America
#4	Avantor (VWR)	Rosny-sous-Bois cedex, France	97068-085	141K20	USA
#5	Biosera	Cholet, France	FB-1280/500	S00AV10004	EU
#6	Biowest	Nuaillé, France	S1400-500	S00F610003	French
#7	Biowest	Nuaillé, France	S1810-500	S00H810001	South America
#8	Merck	Darmstadt, Germany	K7524	BCBV8085	Non-USA origin
#9	Eurobio Scientific	Les Ulis, France	CVFSVF00-0U	S72121-1218	South America
#10	Cytiva Hyclone (Thermo Fisher Scientific)	Waltham, MA, USA	SV30160.02	RF20200006	South America
#11	Seradigm (VWR)	Rosny-sous-Bois cedex, France	3100-050	48B16	USA
#12	Gibco (Thermo Fisher Scientific)	Waltham, MA, USA	10270-106	41Q5484K	Brazil

## Data Availability

Requests for further information about resources, reagents, and data availability should be directed to the corresponding author.
